# Supplementary material for: Reef visitors’ observation of assisted coral recovery devices in situ reduces concern about their use
Source: PLoS One. 2024 Nov 8;19(11):e0313345. doi: 10.1371/journal.pone.0313345 (PMC11548783; doi:10.1371/journal.pone.0313345)
Supplement: Attachment — Submitted filename: Review_PONE-D-24-22940.pdf [file pone.0313345.s001.pdf]

Review of the manuscript titled: “Reef visitors’ observation of assisted coral recovery devices in situ reduces concern about their use”

General understanding of the manuscript

The manuscript presents a study on the impact of Assisted Coral Recovery (ACR) devices on visitor experiences and perceptions at the Great Barrier Reef (GBR). The study evaluates the influence of observing ACR devices on overall trip satisfaction, perceptions of reef beauty, concern about the GBR's future health, and concern about ACR devices themselves.

The manuscript claims (i) no significant influence of ACR devices on overall trip satisfaction, visual beauty perceptions, or concern about the GBR’s health, and a reduced concern about ACR devices among visitors who observed them.

These claims are significant for coral reef management and restoration strategies. Understanding how ACR devices affect public perception and experience is crucial for designing effective reef interventions and fostering public support.

Introduction: The authors place their study within the context of existing literature on visitor perceptions of reef interventions and ACR devices. They appropriately reference studies on factors affecting visitor satisfaction and concern about reef health. The literature review is comprehensive but could benefit from a more detailed discussion of specific studies on public perception of similar reef interventions. Highlighting gaps in existing literature and how this study addresses them would strengthen the context.

Materials and methods: The data are extensive, with large sample sizes and detailed statistical analyses. However, some areas could be improved:

- Supporting Claims: The claim that ACR devices do not significantly impact satisfaction or beauty perceptions is well-supported. However, the authors should consider discussing the potential for different visitor demographics or reef conditions to affect these outcomes.
- Additional Evidence: Including more nuanced analyses, such as interaction effects between different variables, could provide a deeper understanding of the data.

Provide, if possible, additional context or examples where the presence of ACR devices might influence perceptions differently, particularly in larger or more altered reef settings.

About the Protocols and Methods: the methods are appropriate, but detailed protocols for specific analyses or observational criteria are not provided. I recommend the authors to consider including a detailed protocol for the observational aspects of ACR device interactions and visitor perceptions. This would aid in replicability and transparency.

The manuscript is well-organized and written clearly. The structure facilitates understanding for both specialists and non-specialists. But, the clarity of the figure could be improved to ensure that the visual data representations are easily interpretable and well-labeled, especially by the non-specialists, for example, by adding some simple results or other types of illustrations.

The manuscript presents valuable insights into the impact of ACR devices on visitor perceptions at the GBR. However, considering the low concern about ACR devices reported by a significant portion of respondents, it may be beneficial for the authors to address and discuss potential biases or limitations in the survey sample or methodology. Furthermore, addressing the broader implications of the findings in relation to larger-scale interventions and different reef settings would enhance the manuscript.

I recommend encouraging the authors to address the comments provided and resubmit a revised version of the manuscript. The study shows significant potential and could contribute valuable knowledge to the field of coral reef management and restoration.
