# Supplementary material for: Reef visitors’ observation of assisted coral recovery devices in situ reduces concern about their use
Source: PLoS One. 2024 Nov 8;19(11):e0313345. doi: 10.1371/journal.pone.0313345 (PMC11548783; doi:10.1371/journal.pone.0313345)
Supplement: Attachment — Submitted filename: Response to Reviewers.docx [file pone.0313345.s002.docx]

**PONE-D-24-22940	PLOS ONE	11 October 2024**

*“Reef visitors’ observation of assisted coral recovery devices in situ reduces concern about their use”*

**Response to Reviewers and Editor:**

On behalf of my co-authors, we thank you for your consideration and careful review of our manuscript ‘*Reef visitors’ observation of assisted coral recovery devices in situ reduces concern about their use’*. We are grateful for the constructive and positive feedback, and we have done our best to address the reviewers’ suggested revisions, as outlined in Table 1 below. Responses to editorial feedback on the journal’s requirements are provided in Table 2, and an updated Financial Disclosure/Funding statement is provided at the end. We appreciate the opportunity to revise and resubmit our manuscript, which we hope is now worthy of publication in PLOS ONE.

Kind regards,

Matt Curnock (*corresponding author*)

**Table 1: Response to reviewers’ comments**

| **Reviewer feedback** | **Author response** |
| --- | --- |
| **Reviewer #1:**  *General understanding of the manuscript:*  The manuscript presents a study on the impact of Assisted Coral Recovery (ACR) devices on visitor experiences and perceptions at the Great Barrier Reef (GBR). The study evaluates the influence of observing ACR devices on overall trip satisfaction, perceptions of reef beauty, concern about the GBR's future health, and concern about ACR devices themselves.  The manuscript claims (i) no significant influence of ACR devices on overall trip satisfaction, visual beauty perceptions, or concern about the GBR’s health, and a reduced concern about ACR devices among visitors who observed them.  These claims are significant for coral reef management and restoration strategies. Understanding how ACR devices affect public perception and experience is crucial for designing effective reef interventions and fostering public support. | Thank you, and we are hopeful that these findings and insights will be useful for coral restoration practitioners and coral reef managers alike. |
| *Introduction*:  The authors place their study within the context of existing literature on visitor perceptions of reef interventions and ACR devices. They appropriately reference studies on factors affecting visitor satisfaction and concern about reef health. The literature review is comprehensive but **could benefit from a more detailed discussion of specific studies on public perception of similar reef interventions. Highlighting gaps in existing literature and how this study addresses them would strengthen the context**. | We thank the reviewer for this feedback. We have added a new paragraph (lines 90-100, in the *Revised Manuscript with Track Changes*) that describes related studies of public perceptions of coral reef interventions, providing additional context and highlighting a gap filled by our *in situ* study. |
| *Materials and methods*:  The data are extensive, with large sample sizes and detailed statistical analyses.  However, some areas could be improved:  - Supporting Claims: The claim that ACR devices do not significantly impact satisfaction or beauty perceptions is well-supported. However, the authors should **consider discussing the potential for different visitor demographics or reef conditions to affect these outcomes.**  - Additional Evidence: **Including more nuanced analyses, such as interaction effects between different variables,** could provide a deeper understanding of the data.  **Provide, if possible, additional context or examples where the presence of ACR devices might influence perceptions differently**, particularly in larger or more altered reef settings. | We thank the reviewer for this feedback.  **Re: “…consider discussing the potential for different visitor demographics or reef conditions to affect these outcomes.”** – We agree that such factors could indeed influence respondents’ perceptions. We have updated our analysis, adding gender (binary categories only) to our ordinal regression test results, and have updated our Results section, including Fig 3. We found that gender was *not* a significant predictor for any of the response variables.  **We note that when inserting or removing one or more predictor variables from an ordinal regression test, small changes in the resulting regression coefficients for all predictor variables can be expected. Updates have been made through the results section to report the new test results. This has not affected the main findings of the study.**  We were unable to include additional demographic factors, nor investigate the potential effect of different reef conditions due to our study’s limited scope. We have noted these limitations and discussed the potential for these factors to influence visitor perceptions in the discussion section, under the new heading ‘*Limitations, knowledge gaps and further research’* (see lines 498-505, in the *Revised Manuscript with Track Changes*).  **Re: “Including more nuanced analyses, such as interaction effects between different variables...”** – Our primary analysis utilises ordinal logistic regression tests that determine the likelihood that an ordered, categorical response variable can be explained by variation in the predictor variables. The ordinal regression model assumes that the effect of the predictor variables on the response variable is the same for all categories of the response variable. The predictor variables in our analysis were predominantly continuous and not binary, except for gender. While modelling interactions between different variables is possible, adding interaction terms to the models complicates the interpretation of the predictor coefficients, without adding significant value to the main findings. We argue that the ordinal regression tests we have applied are appropriate and rigorous, and that exploring additional interaction effects between predictors was beyond the aims and scope of our study.  **Re: “Provide, if possible, additional context or examples where the presence of ACR devices might influence perceptions differently…”** – We have provided additional context in the Discussion section (see lines 492-497 in the *Revised Manuscript with Track Changes*) about the potential for different reef visitor perceptions to arise in response to ACR deployments at different scales and in different environmental conditions, as well as the potential for other demographic and cultural factors to influence reef visitor perceptions.  We have noted that tour operators’ accompanying interpretive information can potentially also influence visitor perceptions of ACR devices (lines 471-484 in the *Revised Manuscript with Track Changes*). From our review of the literature, we are unaware of any other *in situ* studies of visitor perceptions of ACR devices, in the GBR or elsewhere, that would enable a direct comparison with our findings. |
| *About the Protocols and Methods*:  the methods are appropriate, but detailed protocols for specific analyses or observational criteria are not provided. **I recommend the authors to consider including a detailed protocol for the observational aspects of ACR device interactions and visitor perceptions**. This would aid in replicability and transparency. | In our ‘*Limitations, knowledge gaps and further research section’* (lines 503-505 in the *Revised Manuscript with Track Changes*) we have noted that it was beyond our ability to quantify or control the environmental conditions and myriad other *in situ* variables that may have contributed to respondents’ experiences and survey responses. Controlling for such variables is likely to be a persistent limitation for *in situ* studies in coral reef environments. |
| The manuscript is well-organized and written clearly. The structure facilitates understanding for both specialists and non-specialists. But, **the clarity of the figure could be improved** to ensure that the visual data representations are easily interpretable and well-labeled, especially by the non-specialists, for example, by adding some simple results or other types of illustrations.  The manuscript presents valuable insights into the impact of ACR devices on visitor perceptions at the GBR. However, considering the low concern about ACR devices reported by a significant portion of respondents, **it may be beneficial for the authors to address and discuss potential biases or limitations in the survey sample or methodology**. Furthermore, **addressing the broader implications of the findings in relation to larger-scale interventions and different reef settings** would enhance the manuscript.  I recommend encouraging the authors to address the comments provided and resubmit a revised version of the manuscript. The study shows significant potential and could contribute valuable knowledge to the field of coral reef management and restoration. | Thank you.  **Re: “the clarity of the figure could be improved…”** – We have presented the results from our ordinal regression tests (Fig 3 – with updated results as discussed above) in a standard plot format that shows the effect sizes (regression coefficient) and standard error for each of the variables tested. Variables that had a significant relationship are indicated in blue (for a positive relationship) or red (for a negative relationship). We note that this presentation format for ordinal regression test results is often used in scientific papers, including those published in PLOS ONE (e.g., <https://doi.org/10.1371/journal.pone.0308252>).  We have made minor updates the legend for Fig 3 (see track changes) to ensure it is unambiguous and concise. We have also reviewed the explanatory text that follows in the Results section (in which we report all the individual test results), and we believe this is clear and comprehensive. We are confident that our presentation of the results in this format meets widely accepted academic standards, including those of PLOS ONE.  **Re: “…address and discuss potential biases or limitations in the survey sample or methodology.”** – In our ‘*Limitations, knowledge gaps and further research section’* (lines 498-502, in the *Revised Manuscript with Track Changes*) we have noted that “Our study was limited in scope, recruiting only English-speaking respondents, and using a limited number of survey questions to enable short completion times and minimise its potential intrusiveness for Reef visitors. The GBR receives a diversity of international visitors each year, and future studies that include non-English speaking participants may identify other demographic and cultural factors that influence reef visitor perceptions of ACR devices.”  **Re: “…addressing the broader implications of the findings in relation to larger-scale interventions and different reef settings…”** – We believe we have addressed this adequately in the manuscript, including recognition of potentially different visitor responses to larger scale deployments (lines 493-497 in the *Revised Manuscript with Track Changes*), and we have recommended that further research accompanies different types of intervention at varying scales, including the use of longitudinal studies (lines 506-509 in the *Revised Manuscript with Track Changes*). After reviewing the now-expanded discussion section, we do not consider further elaboration of these points is necessary. |
| **Reviewer #2:**  The manuscript clearly highlights that the use of assisted coral recovery (ACR) is minimally disruptive to visitors’ experiences in the Great Barrier Reef (GBR), and may even contribute to increased public acceptance and support for reef restoration projects. Given the rapid expansion of ACR initiatives due to the urgency to address coral reef degradation, the authors demonstrate the existing opportunity to balance ecological interventions and still maintain the quality of visitor experiences. The findings are well supported by the empirical approach employed, comprehensive range of perspectives drawn from an adequate sample size, and regression analysis which adds rigor to the findings. | Thank you for this positive feedback. |
| The authors' hypothesis focused on visitors' concerns regarding Assisted Coral Recovery (ACR) devices and was framed around potential negative reactions or apprehensions from visitors when they observed these devices. **The study apparently overlooked the potential positive responses, such as appreciation, curiosity, or even admiration and support for conservation efforts. Exploring both sides would have provided a more balanced view of how ACR devices are perceived** and could inform more nuanced conclusions about visitor experiences and their support for coral conservation efforts. In addition, **this manuscript would have benefited from a global representation on public perceptions if the authors moved beyond selecting English-Speaking participants only**, possible by providing translations, given the diversity of international visitors in the GBR. | **Re: “The study apparently overlooked the potential positive responses…”** – While our study aims clearly state that we investigated whether the observation of ACR devices had any effect (positive or negative) on Reef visitor experiences and perceptions (lines 181-184 in the *Revised Manuscript with Track Changes*), we acknowledge that an underlying driver of our study was indeed a concern about the potential for ACR devices to detract from visitor experiences at Reef sites. We note that our survey preamble and questions were framed in a neutral manner (as outlined in the Materials and Methods section; see also the survey instrument that accompanies the full dataset: <https://doi.org/10.25919/7353-m763>), allowing for the detection of positive as well negative relationships between the dependent and predictor variables.  We agree that positive responses such as appreciation, curiosity, admiration and support for conservation are worthy of further investigation, particularly when factors such as the content and delivery of interpretive information can be accounted for in the analysis. Unfortunately, this was beyond our study’s limited scope. We discuss the potential for such benefits to be investigated in the Discussion section (lines 477-486 in the *Revised Manuscript with Track Changes*).  **Re: “…this manuscript would have benefited from a global representation on public perceptions…”** – We acknowledge this limitation of our study and have noted it in the new section ‘Limitations, knowledge gaps and further research’ (see lines 498-502 in the *Revised Manuscript with Track Changes*). |
| Other than that, there were the following minor observations:  1. Page 12, para 1: The decision to use a 10-point Likert scale should be justified, as it seems too coarse particularly over a 5-point or 7-point scale.  2. Page 17, para 1: The analysis shows that respondents who were residents to the GBR were significantly more likely to express a lower level of concern on ACR. However, younger age category showed more concern on ACR observations. The implication of these findings should be emphasized in the discussion, especially on the potential to raise local opportunities for the promotion of ACR interventions. | **Re: point 1 –** We have added a new paragraph explaining our rationale for using 10-point Likert scales in the Materials and Methods section (lines 268-273 in the *Revised Manuscript with Track Changes*).  **Re: point 2 –** The reviewer is referring to results for ‘*Concern about the use of ACR devices’*, also illustrated in Fig 3 panel d. In pointing this out, the reviewer has identified an error that we made in our descriptive text. The negative regression coefficient in fact indicated that *older* (not younger) respondents were slightly more likely to give lower ratings of concern. However, after we re-ran our regression tests to include *gender* among the predictor variables (in response to Reviewer 1’s feedback above), the result for respondent *age* was longer statistically significant.  **We note that when inserting or removing one or more predictor variables from an ordinal regression test, small changes in the resulting regression coefficients for all predictor variables can be expected. Updates have been made through the results section to report the new test results. This has not affected the main findings of the study.**  We thank Reviewer 2 for drawing our attention to that error. Considering the now non-significant test result for age, we do not consider that expanding on this finding in the discussion is warranted. |

**Table 2: Response to editorial feedback**

| **Editorial feedback (journal requirements)** | **Author response** |
| --- | --- |
| 1. Please ensure that your manuscript meets PLOS ONE's style requirements, including those for file naming. | We have updated the manuscript in accordance with PLOS ONE’s style requirements, including the referencing style and titles for display items. File names have also been updated as required. |
| 2. Please note that PLOS ONE has specific guidelines on code sharing for submissions in which author-generated code underpins the findings in the manuscript. In these cases, all author-generated code must be made available without restrictions upon publication of the work. | This is not applicable for our manuscript (i.e. no author-generated code arising from the study). |
| 3. In the ethics statement in the Methods, you have specified that verbal consent was obtained. Please provide additional details regarding how this consent was documented and witnessed, and state whether this was approved by the IRB. | We note that respondents’ consent to participate in the study was not directly recorded on the survey instrument (or by other means).  As described in the Materials and Methods section, prior to questionnaire distribution, an announcement was made to passengers (with the approval of company management and usually delivered by the vessel crew) in which the study was introduced and briefly described, and it was clearly stated that participation was voluntary. Researcher protocols required that all respondents were asked politely if they would like to participate, and they were only given a questionnaire copy if they responded affirmatively. This process was overseen by the vessel crew, who had a strong interest in ensuring that the research was not intrusive and did not detract from passengers’ experiences.  Additionally, information on the questionnaire coversheet specified that respondents were “free to withdraw by stopping the survey at any time”. Consent to participate was implied if respondents completed and returned their questionnaire to the researcher on board. We note that approximately 50 incomplete, returned questionnaires were collected, that were subsequently excluded from the study.  The above researcher protocols and the information provided on the questionnaire coversheet were reviewed and approved by the CSIRO Social Science and Human Research Ethics Committee on 11 August 2023; and were thus deemed ethically appropriate and in accordance with the Australian [National Statement on Ethical Conduct in Human Research](https://www.nhmrc.gov.au/research-policy/ethics/national-statement-ethical-conduct-human-research).  We have added additional minor details to the Methods section in the manuscript (lines 245-247 in track changes) outlining these terms for consent. |
| 4. Please state what role the funders took in the study. If the funders had no role, please state: ""The funders had no role in study design, data collection and analysis, decision to publish, or preparation of the manuscript.""  If this statement is not correct you must amend it as needed.  Please include this amended Role of Funder statement in your cover letter; we will change the online submission form on your behalf. | We note that one co-author is an employee of one of the study’s funding organisations (J. Loder; Great Barrier Reef Foundation), who contributed to the study’s conceptual design and preparation of the manuscript. This co-author was not directly associated with the project’s funding or its administration and was not involved in the data collection and analysis, nor in the decision to publish the manuscript.  **An updated financial disclosure/funding statement is provided below this table (p.10).**  We have also removed funding information details from our Acknowledgements section at the end of the revised manuscript. |
| 5. Please expand the acronym “RRAP ENG” (as indicated in your financial disclosure) so that it states the name of your funders in full.    This information should be included in your cover letter; we will change the online submission form on your behalf. | In the updated financial disclosure statement (below) we have spelled out the RRAP acronym in full, and we have removed the ENG acronym. |
| 6. We note that Figure 2 in your submission contain [map/satellite] images which may be copyrighted. All PLOS content is published under the Creative Commons Attribution License (CC BY 4.0), which means that the manuscript, images, and Supporting Information files will be freely available online, and any third party is permitted to access, download, copy, distribute, and use these materials in any way, even commercially, with proper attribution. For these reasons, we cannot publish previously copyrighted maps or satellite images created using proprietary data, such as Google software (Google Maps, Street View, and Earth). For more information, see our copyright guidelines: http://journals.plos.org/plosone/s/licenses-and-copyright.    We require you to either (1) present written permission from the copyright holder to publish these figures specifically under the CC BY 4.0 license, or (2) remove the figures from your submission | The map shown in Fig 2 was produced using ESRI ArcGIS Pro 3.2.1. All base maps are from the ESRI catalogue. ESRI Terms of Use state the publication of ArcGIS Online maps hosted by ESRI in academic publications (including research journals) is permitted (see: <https://doc.arcgis.com/en/arcgis-online/reference/static-maps.htm#ESRI_SECTION1_21347CA4FAB14A7E95CE6B738DCA2843>). We have updated the legend for Fig 2 with the appropriate citation and have cited the source material in the References [34]. |
| 7. Please review your reference list to ensure that it is complete and correct. If you have cited papers that have been retracted, please include the rationale for doing so in the manuscript text, or remove these references and replace them with relevant current references. Any changes to the reference list should be mentioned in the rebuttal letter that accompanies your revised manuscript. If you need to cite a retracted article, indicate the article’s retracted status in the References list and also include a citation and full reference for the retraction notice. | We have reviewed our reference list, updated the style to PLOS ONE requirements, and we are confident that it is complete and correct.  **Changes to the reference list:**  #23 – In response to feedback from Reviewer 1 (re: including a more detailed discussion of specific studies on public perception of similar reef interventions) we have added a citation to an international case study [23] Ng et al. 2023.  #32 – We had previously cited [32] Whitman et al. 2024 as a preprint. It is now published and we have added full details of the journal and volume number.  #34 – New reference added [34] ESRI 2024, cited in the figure legend for Fig 2.  #40 – New reference added [40] Preston & Colman 2000, cited in Methods section in response to feedback from Reviewer 2 (outlined in Table 1 above).  #41 – New reference added [41] Dawes 2008, cited in Methods section in response to feedback from Reviewer 2 (outlined in Table 1 above).  #50 – We have added a new citation and reference to the Great Barrier Reef Outlook Report 2024, which was published in August 2024. This is the most contemporary, comprehensive and authoritative source of information on the state and trend of values within the Great Barrier Reef Marine Park.  The manuscript does not contain any citations to papers that have been retracted. |
| **Additional Editor Comments:**  Kindly go through the Reviewers comments and address same. | We have done our best to address the Reviewers’ comments, as documented in Table 1 above.  Thank you! |

**Financial disclosure/funding statement (amended):**

This study was conducted as part of research activities by the Reef Restoration and Adaptation Program’s (RRAP) *Best Practice Stakeholder and Traditional Owner Engagement Subprogram* (https://gbrrestoration.org/program/engagement/), with funding provided by the partnership between the Australian Government’s Reef Trust and the Great Barrier Reef Foundation, delivered in partnership with CSIRO, the Great Barrier Reef Marine Park Authority, and the Queensland Government’s Reef Water Quality Program. We note that one co-author is an employee of one of the study’s funding organisations (J. Loder; Great Barrier Reef Foundation), who contributed to the study’s conceptual design and preparation of the manuscript. This co-author was not directly associated with the project’s funding or its administration and was not involved in the data collection and analysis, nor in the decision to publish the manuscript.
